# Using a facile method to predict properties of recycled waste nitrile rubber (NBR) through devulcanization

**DOI:** 10.1038/s41598-023-42438-x

**Published:** 2023-09-16

**Authors:** Mohammad Amin Ghowsi, Masoud Jamshidi

**Affiliations:** Constructional Polymers and Composites Research Lab., School of Chemical, Petroleum and Gas Engineering, Iran University of Science and Technology (IUST), Tehran, Iran

**Keywords:** Pollution remediation, Chemical engineering, Polymers

## Abstract

To prepare a reliable method for predicting the properties of devulcanized rubbers a nitrile rubber (NBR) compound was prepared and masticated before vulcanization for 0, 30 and 60 min under mechanical stress to prepare NBRs with different molecular weights. The masticated samples were vulcanized at different accelerator contents to prepare damples with different crosslink densities. The physical/mechanical/thermal properties (i.e. crosslink density, tensile strength, modulus, modulus at 100 and 300% elongation, elongation at break, hardness, curing behavior and molecular weight) of the samples were experimentally evaluated. In the next step, the prepared samples were assumed as devulcanized NBRs that underwent chains scission (masticated samples) or crosslinks breakage (vulcanized at different accelerator contents). On this basis, hypothetical devulcanization routes were considered between each sample that underwent chains scission or crosslinks breakage. Based on the results, numerical relationships between the number of chains scission or crosslinks breakage and decrease in the properties were obtained. Finally, the numerical reationships were used to calculate the properties of the samples that underwent both of chains scission and crosslinks breakage. It was found that the calculated contents of hardness, modulus at 100% and molecular weight (M_Z_) using the prepared method were very close to the evaluated ones.

## Introduction

Recycling of waste rubbers is of the great interests of researchers due to environmental, conservation of raw materials and economical aspects^1^. Devulcanization is a promising method among other waste rubber recycling methods^2,3^. During devulcanization process, crosslinks and polymer chains are broken that enable it to flow and mold again. On this basis, the devulcanized rubber could be re-vulcanized and reused^4^. Practically, breaking of all crosslinks is impossible and fracture in the polymer backbone also occurs at the same time that this lowers the mechanical properties of recycled rubber^5,6^. In fact, the key factor for successful devulcanization of a waste rubber is increasing in the crosslinks breakage and lowering the chains scission^7^. This makes devulcanization method a smarter recycling process than reclamation.

In the last decades, many researchers focused on assessing the quality of recycled rubbers through devulcanization process. Hirayama et al.^8^ measured the quality of devulcanized SBRs containing different contents of carbon black through microwave irradiation. They evaluated sol/gel contents of the recycled rubbers and conducted TGA and DMTA analysis on the samples. It was found that carbon black content has a great impact on devulcanization content by microwave irradiation. Ghowsi et al.^9^ devulcanized waste nitrile rubber powder using different chemical agents. They mechanically devulcanized rubber particles on a two roll mill. The sheet formation time from particles was considered as a criterion for devulcanization performances. It was found that the sheet formation time (i.e. devulcanization content) decreases about 50 and 85% in presence of different devulcanizing agents and heating, respectively. Rios et al.^10^ devulcanized SBR with a controlled oxidation process via nitric acid. They evaluated the success of the process using IR, CNMR, TG, GPC and TPD-MS analysis. Rooj et al.^11^ devulcanized NR rubber in presence of benzoyl peroxide as a chemical devulcanizing agent and monitored the process by measuring crosslink density, tensile properties and IR and SEM analysis. They showed that chemo-mechanical devulcanization is more efficient than separate chemical and mechanical methods. Sabzekar et al.^12^ studied devulcanization of EPDM rubber in presence of a disulfide oil as chemical agent. They used chemo-mechanical devulcanization method and monitored the process by measuring crosslink density (CLD), sol/gel contents, hardness, tensile properties, compression set and resilience of the recycled rubbers. It was found that the best results obtained with 7 phr of disulfide oil at a temperature of 290 °C and screw speed of 120 rpm. The decrease in the CLD and increase in the sol content were introduced as critera for choosing the best devulcanization process. They also blended devulcanized EPDM with virgin rubber at different ratios and claimed that the product could be industrially attractive based on the physical and mechanical properties.

Vahdatbin et al.^13^ devulcanized NR/SBR waste with various devulcanizing agents at different times of MW irradiation. They evaluated qualitatively/quantitatively devulcanization of samples using evaluation of crosslink density before and after devulcanization. They also investigated the effect of the size of waste rubber particles on the devulcanization performances. They claimed that waste rubber particles were partially devulcanized (i.e. devulcanization of the shell of particles) and devulcanization content improved by decrement in the particle size of the particles. Shabani et al.^14^ devulcanized NR/SBR waste by probe sonication and assessed the success of process using determination of sol/gel contents and crosslink densities before and after devulcanization. They also claimed that partial devulcanization occurred through probe sonication that its performance depend on the sonication power/time and chemical agent type. Molanorouzi et al.^15^ used different chemical agents for devulcanization of waste tire rubber. The Horikx theory was used to determine the success of devulcanization process for breaking the crosslinks instead of chains scission.

Based on the literature survey, different methods have been conventionally used for qualitative/quantitative evaluation of devulcanization process. For instance, measurment of sol/gel contents and crosslink density^16,17^, rheological properties^18^ and mechanical features^19^ have been performed for this purpose, hitherto. Furthermore, some analysis such as CNMR^12^, GPC^20^, FTIR^20^, TGA^21^, SEM^22^, DSC^23^, DMA/DMTA^24^ have been used to characterize devulcanized rubbers. Some researchers also used Horikx theory for this purpose^25^.

Although, these methods are helpful for evaluation of devulcanization process (i.e. quality of the recycled rubber) but due to the complexity of the process, they are not able to provide a precise explanation on the mechanism of devulcanization, content of crosslinks breakage and chains scission and their relationships to the recycled rubber properties. In fact, most of the efforts have been focused on the optimization of the devulcanization process to achieve higher mechanical properties and devulcanization percentages (or lower crosslink density). The Horikx theory is the lone method that has been used to illustrate the success of crosslinks breakage in devulcanization process that is performed based on statistical calculations^25,26^. However, preparing Horikx curve is time-consuming and expensive. Besides, it could not be used for partially devulcanized rubber^9^.

There are few investigations on evaluation of the crosslinks breakage and chains scission during devulcanization process and their impact on the physical/mechanical properties of the recycled rubber. In fact, it seems to be impossible to separate role of crosslinks breakage and chains scission on the properties of the devulcanized process using the traditional methods.

In this work, it was aimed to prepare a method to separate effect of crosslinks breakage and chains scission on decrement in the properties of a nitrile rubber during devulcanization. For this purpose, samples with different molecular weights (but the same formulation) as a simulated devulcanized samples that exposured just to the chains scission were prepared, at first. The mechanical stress (mastication) was applied for three different times (i.e. 0, 30 and 60 min) to a masterbatch of NBR (uncured). On this basis, numerical relationships were obtained between the chains scission and the decrement content in the properties of the masticated samples. In the second step, the prepared samples with different chain lengths (molecular weights) were vulcanized at various contents of CBS accelerator (i.e. low, medium and high contents). In this way, three category of materials with different molecular weight were prepared that each category contains three samples with different crosslinking contents. In this case, each category could be assumed as the samples with different content of crosslinks breakage without any chains scission. Now, we have three types of samples that exposed just to chain scission, crosslinks breakage or both of them during hypothetical devulcanization processes. The numerical relationship between the crosslink breakage and the decreased properties was achieved. The physical/mechanical properties of the prepared samples (i.e. curing behavior, tensile properties, hardness, crosslink density, devulcanization percent and molecular weight) were considered in this study. Finally, based on the relationships between the decreased content of the properties and number of chains scission or crosslinks breakage, the method was used to predict the properties of the samples that experienced both chains scission and crosslinks breakage during devulcanization. Furthermore, the devulcanization mechanism also was studied using Horikx theory for comparison.

## Experimental

### Materials

The raw materials used in this research are listed in Table 1.

**Table 1:** The used raw materials in this study.

Materials	Producer (Country)
NBR grade 6240	LG chemicals Co. (South Korea)
DOP (Di Octyl Phthalate)	LG chemicals Co. (South Korea)
C9 Petroleum resin grade GA120	Yuen Liang Co. (Taiwan)
Zinc oxide	Kian Rouy Co. (Iran)
Rubber grade stearic acid	KLK Co. (Malaysia)
Carbon black N660	Doodeh Sanati Pars Co. (Iran)
Calcium carbonate grade omyacarb5 SW	Omya Pars Co. (Iran)
Granular paraffin wax	Rose Polymer Co. (Iran)
IPPD or 4010NA	Dalian Richon Chem Co. (China)
Sulfur grade SU95	Schill + Seilacher Co. (Germany)
CBS (n-cyclohexyl -2- benzothiazole sulfenamide)	Dalian Richon Chem Co. (China)
TMTD (Tetramethylthiuram disulfide)	Dalian Richon Chem Co. (China)

### Preparation of NBR samples

The used recipe for preparation of NBR compound is given in Table 2. The ingredients were introduced to an internal mixer with a working volume of 10 L. After complete mixing for 10 min, one kilogram of the sample was removed from internal mixer (i.e. category of ${M}_{0}$) and the remained compound was exposed to excess mechanical stresses (i.e. mastication) for 30 min. At this time, one kilogram of the sample was removed from mixer (i.e. category of ${M}_{30}$). The mixing was then continued until 60 min (i.e. category of ${M}_{60}$). The samples were left for 24 h for stress relaxation after mastication and then milled on a two-roller mill to prepare 2 mm rubber sheets.

**Table 2:** Formulation of the prepared NBR compounds.

Raw materials	Phr
Nitrile rubber (NBR 6240)	100
Dioctyl phthalate (DOP)	35
Petroleum Resin (GA120)	2
Zinc Oxide	5
Stearic Acid	0.5
Carbon Black (N660)	67
Calcium Carbonate	50
Paraffin Wax	2
N-Isopropyl-N’-phenyl-1,4-phenylenediamine (IPPD)	1
SUM	262.5

**Figure 1:**
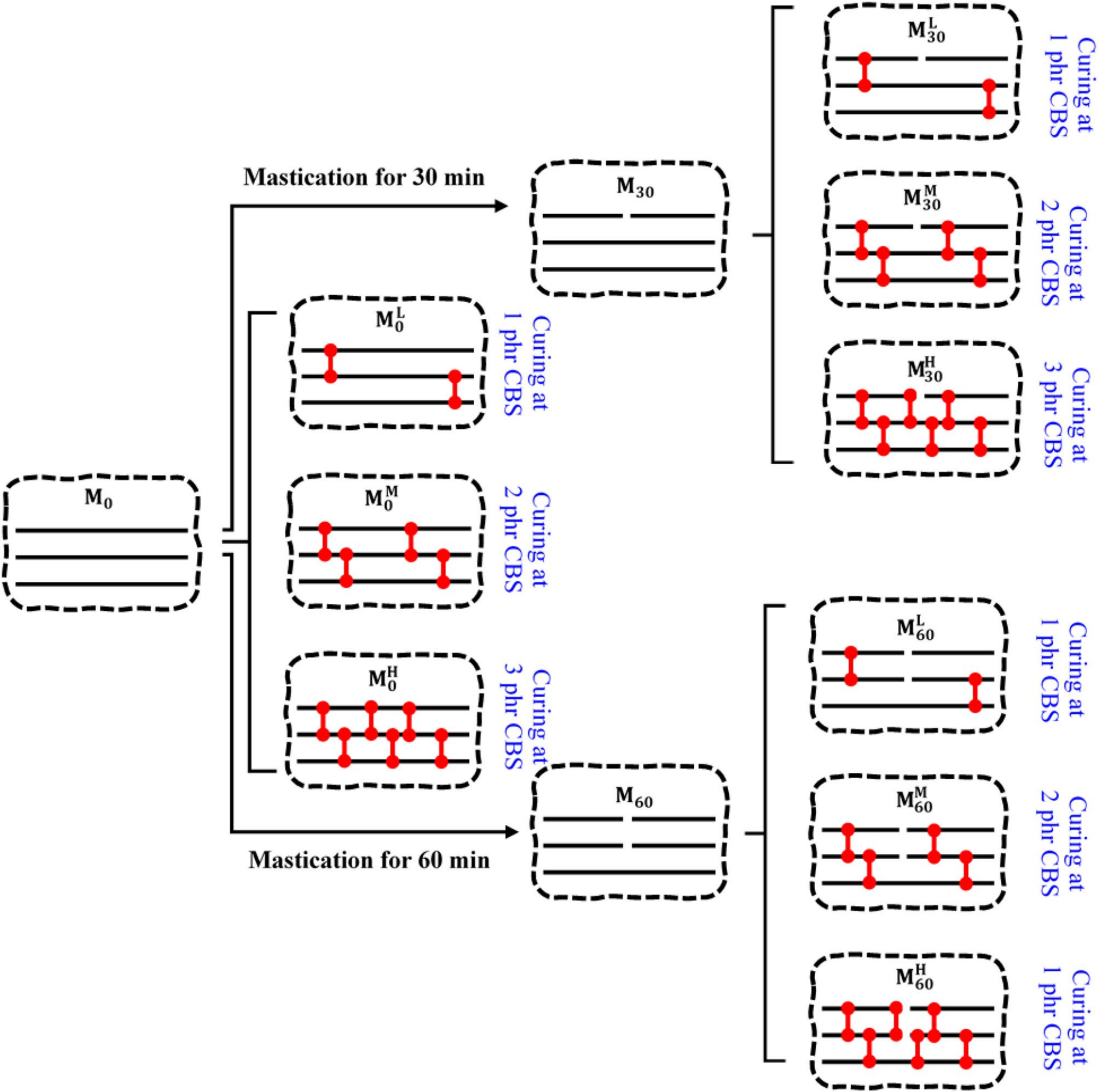
Scheme for the prepared NBR samples based on the mastication times and crosslink densities.

The control samples (i.e., M_0_, M_30_ and M_60_) were mixed with different contents of CBS accelerator (i.e., 1, 2 and 3 phr) at constant sulfur content (i.e. 1.3 phr) and TMTD (0.8 phr) as curing system. Different CBS contents were used to prepare samples with low (L), medium (M) and high (H) crosslink densities. All the samples were cured at 170 °C for 6 min. On this basis, nine samples in three categories of molecular weights (i.e., M_0_, M_30_ and M_60_) and three type of crosslink densities (M^L^, M^M^ and M^H^) were prepared. Figure 1 shows a scheme for the prepared samples.

### Simulating devulcanization of NBR samples

In this research, a creative method was used for monitoring devulcanization process of waste nitrile rubber. According to Fig. 1, hypothetical devulcanization routes were considered for sample pairs (e.g. preparing ${M}_{60}^{L}$ by devulcanization of ${M}_{30}^{H}$). On this basis, 27 hypothetical devulcanization routes were established for different sample pairs.

### Methods

–Calculations

The method for calculation of average molecular weight (M_Z_) of the samples is presented in supporting information, section "Introduction"S.

The crosslink density (CLD) of the samples was measured using the Flory-Rehner relationship^13^ as follows:

1 $$CLD = \frac{{ - \left[ {{\text{ln }}\left( {{ }1 - V_{r} } \right) + V_{r} + V_{r}^{2} \times {\upchi }_{1} } \right]}}{{V_{s} \left( {V_{r}^{\frac{1}{3}} - {\raise0.7ex\hbox{${V_{r} }$} \!\mathord{\left/ {\vphantom {{V_{r} } 2}}\right.\kern-0pt} \!\lower0.7ex\hbox{$2$}}} \right)}}$$

where CLD (mol/m^3^), V_r_ (no unit), V_s_ (m^3^/mol) and χ_l_ (dimensionless) were crosslink density, rubber volume fraction in the swollen sample, molar volume of the solvent and the Flory–Huggins' interaction parameter, respectively. The value of ${\chi }_{1}$(i.e. 0.514 ) for NBR 33% was obtained by averaging the ${\chi }_{1}$ values for NBR 30 and 39% at 25 °C^27^.

To measure crosslink density of the NBR samples, they were extracted in isopropanol solvent according to ASTM D6814^28^. The extraction was performed before swelling to measure the crosslink density. The isopropanol extraction was done according to the ASTM D297^29^. After isopropanol extraction, all the samples were immersed in a sufficient amount of benzene solvent for 72 h. Solvents were refreshed every 24 h. Then the weight of the swollen samples was measured with an accuracy of 0.1 mg. Thereafter, the samples were dried at temperature of 70 ± 2 °C for 24 h in a ventilated oven. The weight of the dried samples was measured with an accuracy of 0.1 mg after reaching to ambient temperature. The value of $V_{r}$. was calculated from Eq. (2):

2 $$V_{r} = \frac{{\frac{Weight\,of\,dried\,rubber\,in\,gr}{{Density\,in \frac{gr}{{cm^{3} }}}}}}{{\frac{Weight\,of\,dried\,rubber\,in\,gr}{{Density\,in \frac{gr}{{cm^{3} }}}} + \frac{{Weight\,of\,solvent\,\left( {benzene} \right)\,absorbed\,in\,gr}}{{Density\,of\,solvent\,\left( {benzene}\,\right)in\,\frac{gr}{{cm^{3} }}}}}}$$

The density of the dried samples was measured in methanol according to ASTM D297 as follows:

3 $${\text{Density}}\;{\text{at}}\;23^{\circ}{\text{C}}\;{\text{in}}\;\frac{{{\text{gr}}}}{{{\text{cm}}^{3} }} = 0.7913 \times \frac{{Sample\;weight\;in\;air\;in\;grams}}{{Sample\;weight\;in\;air\;in\;grams - sample\;weight\;in\;methanol\;in\;gram}}$$

Based on the results, devulcanization percent of the samples were determined according to ASTM D6814 as follows:

4 $${\text{Devulcaniztion}}\% = \left\{ {1 - \left( {\frac{Crosslink\;density\;of\;devulcanized\;rubber}{{Crosslink\;density\;of\;the\;control\;crumbed\;rubber}}} \right)} \right\} \times 100$$

The average molecular weight between two crosslinks $\left( {M_{c} } \right)$ was also obtained using Eq. (5):

5 $$M_{c} = { }\frac{1}{{\frac{CLD}{{density}} + \frac{2}{M}}}$$

where M and density are the average molecular weight of the polymer before crosslinking and density of dried sample (see Eq. 3)^30^.

–sts and analysis

A moving die rheometer (MDR) was used to evaluate curing behavior and rheology of the samples. The test was performed at 170 °C by a Hiwa MDR rheometer instrument. The curing rate index (CRI) was calculated based on the MDR results as following:

6 $${\text{CRI}}\left( \% \right) = 100/\left( {tc90 - ts2} \right)$$

where t_c90_ and t_s2_ are time of reaching of compound to its 90% of curing level and scorch time.

The tensile tests were performed on the cured samples (i.e. at 170 °C for 6 min) using a Instron universal machine at tension rate of 500 mm/min based on ASTM D412^31^. The hardness test was performed on the cured samples by a Cori duromerer based on ASTM D2240^32^.

The devulcanization content was calculated for each hypothetical devulcanization route. For this purpose, 10 chains of a sample were considered and the chains scission and crosslinks breakage were determined. These 10 chains were obtained based on a try and error metod that through it the calculated average molecular weight were almost equal to the evaluated one. In fact, cosidering 10 chains gives the closest results for calculated and evaluated average molecular weights.

**Figure 2:**
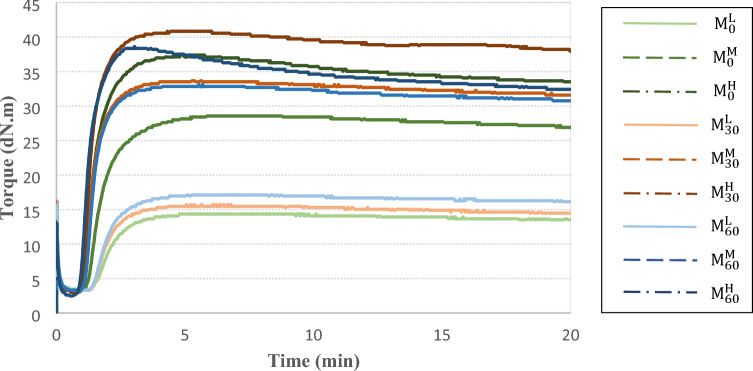
MDR curves of the NBR compounds.

Figure 2S shows scheme of a sample devulcanization route for preparing $M_{60}^{L}$ sample from $M_{30}^{H}$ sample. To calculate devulcanization content of the samples, the number of chains scission and crosslinks breakage that happened through hypothetical route were determined (see section "Experimental"S in supporting information).

Gel permeation chromatography (GPC) analysis was used to characterize molecular weight of the uncured $M_{0}$, $M_{30}$ and $M_{60}$ samples as representatives of their categories. For this purpose, the samples were completely dissolved in THF solvent. The additives were separated using high speed centrifuge. The remaining solution was injected into the GPC analysis column to measure the molecular weight of the dissolved chains.

## Results and discussions

### Properties of the prepared NBRs

**Figure 3:**
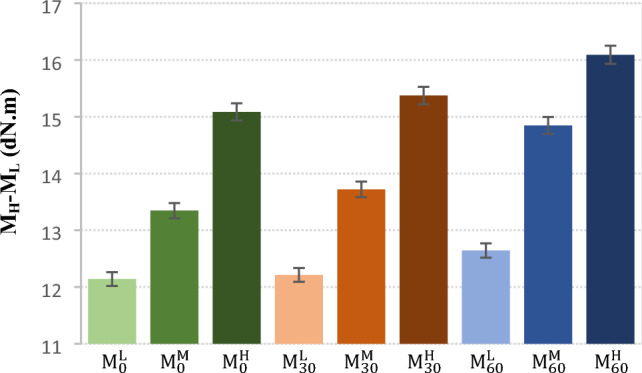
M_H_-M_L_ content of the NBR samples.

The MDR results for NBR compounds are represented in Fig. 2 and Table 3. It is seen that the differences between curing time (t´_90_) of the samples are less than 1.1 min. The curing rate and M_H_-M_L_ content enhanced by increment in the CBS content (see Fig. 3). This confirmed that crosslinking increased by loading more CBS contents. Furthermore, results showed that curing time decreased by 30 min of mastication and this descending trend continued by increment in the mastication time. It was attributed to this fact that some broken chains and radicals have been created during mastication that they could participate in curing process^1^.

**Table 3:** Curing properties of the NBR samples.

Sample code	${t}_{90}{\prime}$ (min)	Ts_2_ (min)	M_L_ (dN.m)	M_H_ (dN.m)
${\mathrm{M}}_{0}^{\mathrm{L}}$	3.1	1.7	1.7	13.9
${\mathrm{M}}_{0}^{\mathrm{M}}$	3.1	1.3	1.5	14.9
${\mathrm{M}}_{0}^{\mathrm{H}}$	2.5	1.1	2.1	17.2
${\mathrm{M}}_{30}^{\mathrm{L}}$	2.9	1.6	1.8	14.0
${\mathrm{M}}_{30}^{\mathrm{M}}$	2.3	1.1	1.7	15.5
${\mathrm{M}}_{30}^{\mathrm{H}}$	2.1	1.0	2.1	17.5
${\mathrm{M}}_{60}^{\mathrm{L}}$	2.8	1.6	2.0	14.7
${\mathrm{M}}_{60}^{\mathrm{M}}$	2.2	1.1	2.0	16.8
${\mathrm{M}}_{60}^{\mathrm{H}}$	2.0	0.9	2.3	18.4

**Figure 4:**
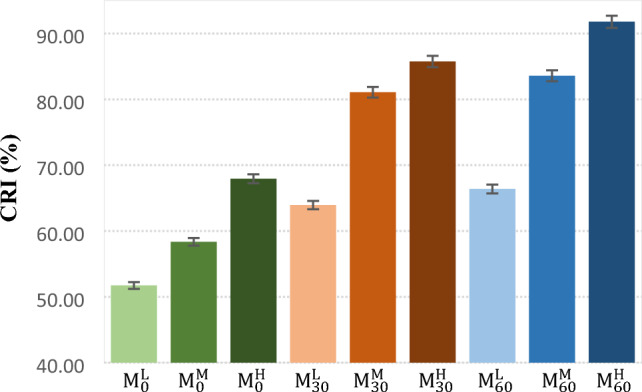
Curing rate index of the NBR samples.

The curing rate index (CRI) of the samples are shown in Fig. 4. It is seen that the curing rate index increased with increasing in the CBS content. It corresponded to creation of more active sites in presence of more CBS in rubber compound during vulcanization. It was also found that the curing rate of rubber samples enhanced by increment in the mastication time at the same CBS content. This was also related to the creation of more free radicals during mastication that could react to sulfur during curing process.

**Figure 5:**
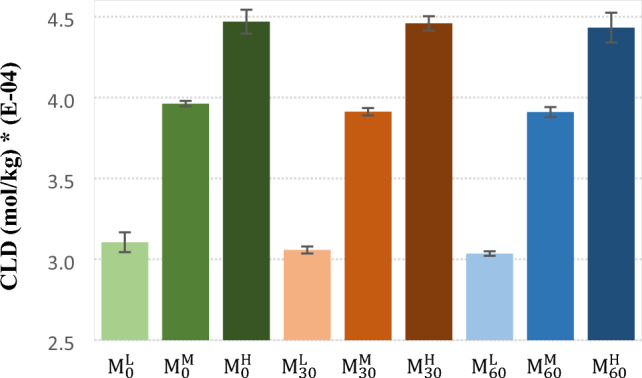
Crosslink density of the cured control and masticated NBR samples.

The crosslink densities (CLDs) of the cured samples were calculated based on the Flory-Rehner equation that results are shown in Fig. 5. Results showed that crosslink density increases with addition of more CBS to different masticated samples. There were negligible differences between the CLDs of the samples at the same CBS contents. It was expected due to this fact that mastication of the raw compound (non-vulcanized) caused just breaks in the chains. On this basis, the CLD is not a suitable criterion for monitoring of the devulcanization process when just chain scission occures.

**Figure 6:**
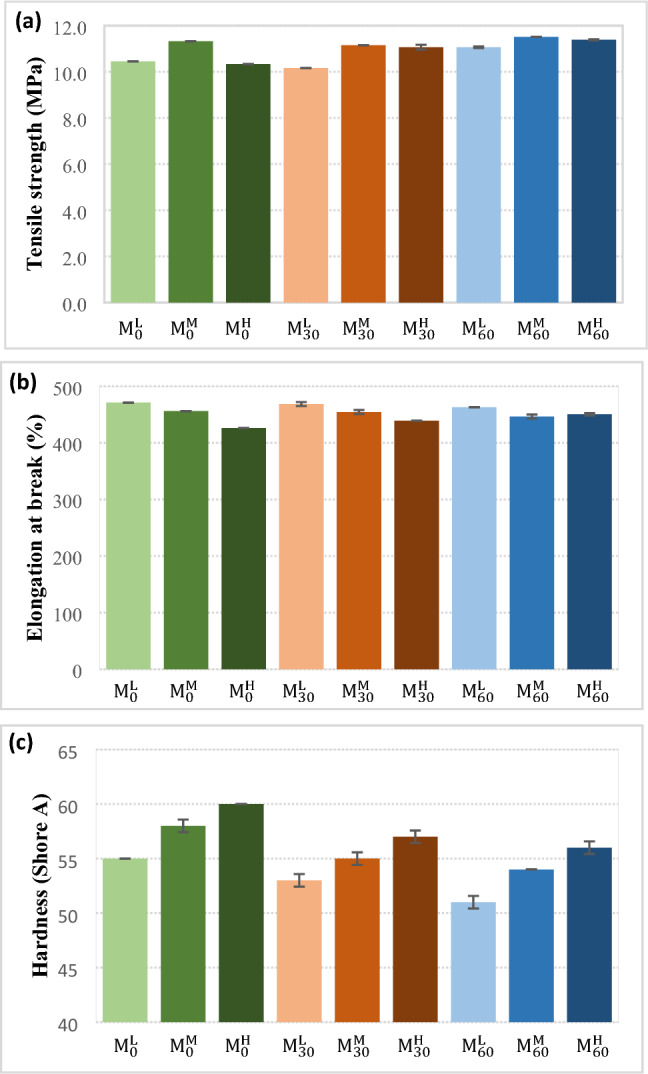
The mechanical properties of the NBR samples; (**a**) Tensile strength, (**b**) Elongation at break and (**c**) Hardness.

Figure 6 shows the mechanical properties of the samples. It is obvious that the tensile strength and elongation at break changed slightly by increment in the mastication time and CBS content (see Fig. 6-a and 6-b). In contrast, hardness of the samples showed considerable changes (see Fig. 6-c). In the samples that did not underdo mastication, the hardness considerably enhanced by increment in the CBS content. It is normal because increased crosslink density at higher CBS content enhances the hardness. The same trend was also seen for the masticated samples (for 30 and 60 min). It was also found that mastication caused decrement in the hardness of NBR samples. Mastication caused chains scission and decrement in the molecular weight that declined hardness of rubber samples.

**Figure 7:**
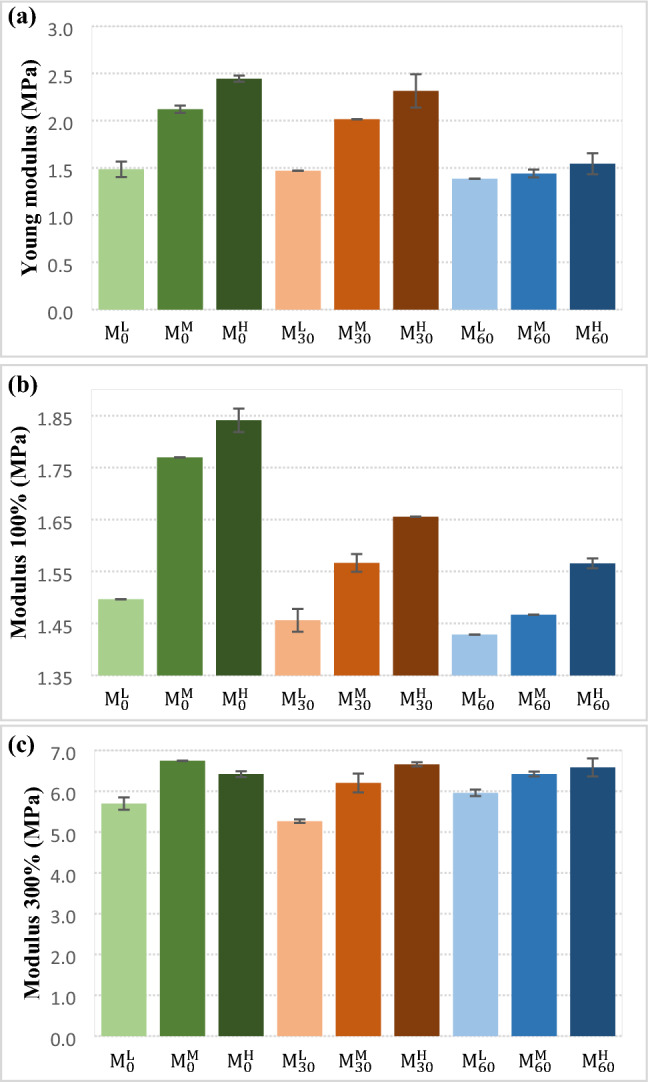
Modulus of the NBR samples; (**a**) Young’s modulus, (**b**) modulus at 100% elongation and (**c**) modulus at 300% elongation.

Figure 7 shows results of Young modulus, modulus at 100 and 300% of the samples. According to Fig. 7-a, it was found that mastication of NBR for 30 min slightly affected the Young modulus of the NBR samples. However, Young modulus declined considerably by more mastication (i.e. 60 min) and it was not compensated by increasing in the crosslink density. In all samples, increasing in the CBS content obviously affected the modulus at 100 and 300%. Generally, Young modulus and modulus at 100 and 300% were increased by increment in the crosslink density.

The mastication of NBR sample caused decrement in the Young modulus and modulus at 100% at different crosslink densities (see Fig. 7-b). However, modulus at 300% was affected significantly by increasing in the crosslink density. In fact, the modulus at 300% of the masticated rubbers were compensated through more crosslinking (see Fig. 7-c).

**Figure 8:**
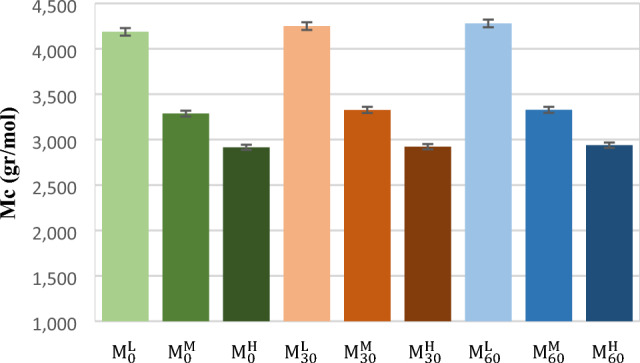
M_c_ content of the cured (i.e.control and masticated) NBR samples.

Figure 8 represents Mc content (i.e., the average molecular weight between two crosslinks) for the NBR samples. It was illustrated that mastication has no effects on Mc of the samples (i.e. like CLD) because Mc shows distance between adjacent sound crosslinks (i.e. crosslinks that were not fractured during mastication). On this basis, Mc could not be a good criterion for predicting devulcanization performance.

**Figure 9:**
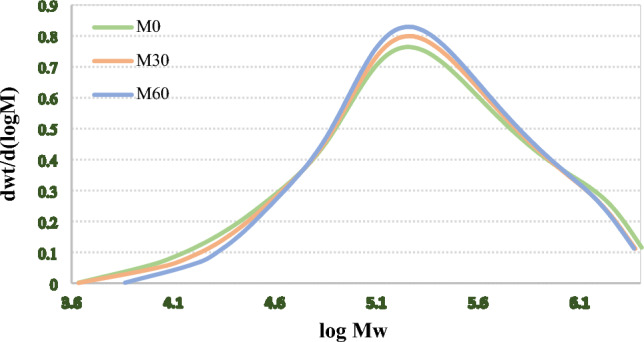
GPC analysis of M_0_, M_30_ and M_60_ compounds.

**Figure 10:**
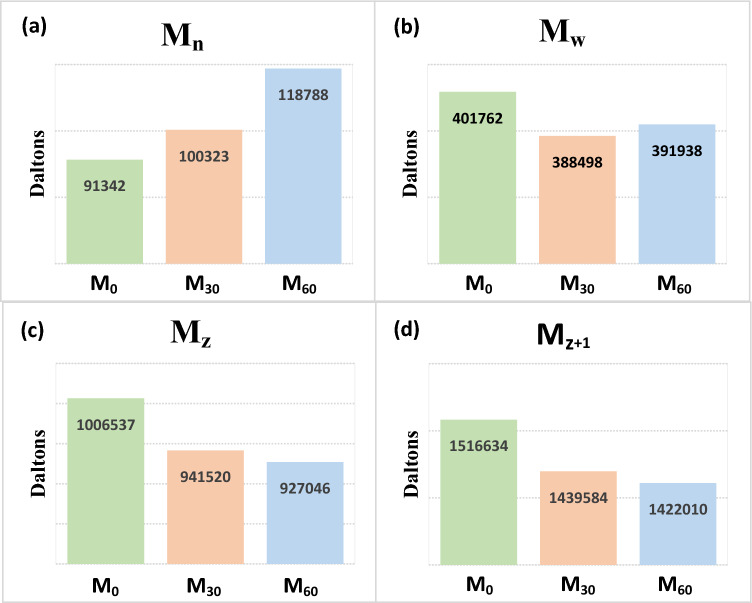
Changes in the molecular weights of the masticated samples; (**a**) M_n_, (**b**) M_w_, (**c**) M_z_ and (**d**) M_z+1_.

GPC analysis was performed to study the molecular weight of the NBR samples. The result are shown in Fig. 9. Based on the GPC results, the molecular weigh values (i.e. M_n_, M_w_, M_z_ and M_z+1_) of the samples were determined (see Fig. 10). According to the results, Mn and Mw values are not reliable data for monitoring the vulcanization performance, because they increased after vulcanization of the samples, while they were expected to decrease due to the decrease in molecular weight occurred by mastication. However, ${\mathrm{M}}_{\mathrm{z}}$ and ${\mathrm{M}}_{\mathrm{z}+1}$ showed reasonable decrement in the molecular weight of the masticated polymers compared to the virgin sample. On this basis, M_z_ and M_Z+1_ are suitable parameters for evaluating the devulcanization perormance. However, M_Z_ is preferred due to its simplicity of calculation compared to M_z+1_.

### Preparation of a method for predicting the properties

To simulate devulcanization process of the NBR samples and find numerical relationships between their properties and devulcanization process, a rubbery matrix contained 10 chains of NBR were considered (i.e. based on the differences between the evaluated M_z_ and calculated M_z_ values) (see Fig. 1S).

Table 4 represents calculated number of crosslinks for the samples. The devulcanization percent for each hypothetical devulcanization route was calculated based on the calculated number of crosslinks. Results are shown in Table 5.

**Table 4:** The calculated crosslinks number for the cured NBR samples (i.e. in a model system with 10 chains of polymer).

Sample	Number of total chains	Number of broken chains	Calculated number of crosslinks^a^
${\mathrm{M}}_{0}^{L}$	10	0	2163
${\mathrm{M}}_{0}^{M}$	10	0	2756
${\mathrm{M}}_{0}^{H}$	10	0	3108
${\mathrm{M}}_{30}^{L}$	10	2	2013
${\mathrm{M}}_{30}^{M}$	10	2	2572
${\mathrm{M}}_{30}^{H}$	10	2	2929
${\mathrm{M}}_{60}^{L}$	10	3	1930
${\mathrm{M}}_{60}^{M}$	10	3	2482
${\mathrm{M}}_{60}^{H}$	10	3	2811

^a^The number of crosslinks were calculated based on the total molecular weight (average of broken and intact chains molecular weights) using equation of (2).

**Table 5:** Calculated devulcanization percent for all hypothetical devulcanization conditions.

Row no	Hypothetical devulcanization		Devulcanization type	Number of crosslinks		Breakage number		Calculated devulcanization percent (CDP) (%)
	before	after		before	after	Backbone	Crosslinks^a^	
1	${M}_{0}^{H}$	${M}_{0}^{L}$	Ideal devulcanization^b^	3108	2163	0	945	30.41
2	${M}_{0}^{M}$	${M}_{0}^{L}$		2756	2163	0	593	21.52
3	${M}_{0}^{H}$	${M}_{0}^{M}$		3108	2756	0	352	11.33
4	${M}_{30}^{H}$	${M}_{30}^{L}$		2929	2013	0	916	31.27
5	${M}_{30}^{H}$	${M}_{30}^{M}$		2929	2572	0	357	12.19
6	${M}_{60}^{H}$	${M}_{60}^{L}$		2811	1930	0	881	31.34
7	${M}_{60}^{H}$	${M}_{60}^{M}$		2811	2482	0	329	11.70
8	${M}_{30}^{M}$	${M}_{30}^{L}$		2572	2013	0	559	21.73
9	${M}_{60}^{M}$	${M}_{60}^{L}$		2482	1930	0	552	22.24
10	${M}_{30}^{L}$	${M}_{60}^{L}$	Worst devulcanization^c^	2013	1930	1	83	4.12
11	${M}_{30}^{M}$	${M}_{60}^{M}$		2572	2482	1	90	3.50
12	${M}_{30}^{H}$	${M}_{60}^{H}$		2929	2811	1	118	4.03
13	${M}_{0}^{M}$	${M}_{30}^{M}$		2756	2572	2	184	6.68
14	${M}_{0}^{L}$	${M}_{30}^{L}$		2163	2013	2	150	6.93
15	${M}_{0}^{H}$	${M}_{30}^{H}$		3108	2929	2	179	5.76
16	${M}_{0}^{L}$	${M}_{60}^{L}$		2163	1930	3	233	10.77
17	${M}_{0}^{M}$	${M}_{60}^{M}$		2756	2482	3	274	9.94
18	${M}_{0}^{H}$	${M}_{60}^{H}$		3108	2811	3	297	9.56
19	${M}_{30}^{M}$	${M}_{60}^{L}$	Usual devulcanization^d^	2572	1930	1	642	24.96
20	${M}_{30}^{H}$	${M}_{60}^{L}$		2929	1930	1	999	34.11
21	${M}_{30}^{H}$	${M}_{60}^{M}$		2929	2482	1	447	15.26
22	${M}_{0}^{H}$	${M}_{30}^{L}$		3108	2013	2	1095	35.23
23	${M}_{0}^{H}$	${M}_{30}^{M}$		3108	2572	2	536	17.25
24	${M}_{0}^{M}$	${M}_{30}^{L}$		2756	2013	2	743	26.96
25	${M}_{0}^{H}$	${M}_{60}^{L}$		3108	1930	3	1178	37.90
26	${M}_{0}^{H}$	${M}_{60}^{M}$		3108	2482	3	626	20.14
27	${M}_{0}^{M}$	${M}_{60}^{L}$		2756	1930	3	826	29.97

^a^The number of breakage in crosslinks = (the total number of crosslinks before breakage) – (the number of crosslinks after breakage).

^b^Due to the breakage just in the crosslinks.

^c^Due to the low breakage in the crosslinks compared to chains scission.

^d^Due to the more breakage in the crosslinks compared to chains scission.

To find numerical relationships between the evaluated properties and the calculated number of the fractured crosslinks in ideal devulcanization condition (i.e. devulcanization routes that represented in rows 1 to 9 of Table 5), the differences in the evaluated properties and calculated crosslinks numbers were determined and listed in Table 6. The results show the number of crosslinks breakage that are required for one unit change in each property in ideal devulcanization condition (i.e., without chains scission) (see Tables 1S to 6S for detailed information).

**Table 6:** The number of crosslinks breakage (CLB) which caused one unit reduction in the properties.

Row no	Number of CLB	Reduction in Evaluated hardness (Shore A)	Number of CLB for 1 Shore A reduction in Hardness	Number of CLB for 1 MPa reduction in Young’s Modulus	Number of CLB for 1 MPa reduction in Modulus at 100%	Number of CLB for 1 MPa reduction in Modulus at 300%	Number of CLB for 1 dN.m reduction in M_H_	Number of CLB for 1 dN.m reduction in M_H_-M_L_
1	945	5	189	985	2742	1310	286	321
2	593	3	198	933	2171	566	598	492
3	352	2	176	1086	4923	-1078	152	202
4	916	4	229	1083	4596	658	266	290
5	357	2	179	1186	4020	783	177	216
6	881	5	176	5565	6426	1410	236	256
7	329	2	165	3207	3330	2020	207	264
8	559	2	280	1026	5059	597	389	371
9	552	3	184	9910	14413	1195	257	251
Standard Deviation	36	3098	3655	860	136	90		
Validated average	187	1884	4158	931	246	280		

According to Fig. 1, the devulcanization percent should be zero for hypothetical routes in the worst devulcanization condition (i.e. the devulcanization routes listed in rows 10 to 18 of the Table 5) due to the mastication (just chain scission) that happened in these routes. Deviations were obtained between the evaluated and calculated crosslinks breakage number (see Table 6) that were corresponded to the errors of measurement method.

Table 7 shows the changes in the properties in the case of worst devulcanization condition (i.e. just chains scission). Based on the results, the numerical relations between number of chains scission and one unite decrement in the evaluated properties were determined.

**Table 7:** The number of chains scission (Cs) which caused one unit decrement in the properties.

Row no	Number of CS	Decrease in hardness (Shore A)	Decrease in Young modulus (MPa)	Decrease in modulus at 100% (MPa)	Decrease in modulus at 300% (MPa)	Increase in M_H_ (dN.m)	Increase in M_H_-M_L_ (dN.m)
10	1	2	0.0847	0.0275	− 0.6940	0.646	0.431
11	1	1	0.5738	0.0997	− 0.2192	1.354	1.126
12	1	1	0.7722	0.0897	0.0737	0.934	0.718
13	2	3	0.1062	0.2031	0.5427	0.589	0.374
14	2	2	0.0154	0.0404	0.4314	0.144	0.072
15	2	3	0.1292	0.1858	− 0.2397	0.288	0.2880
16	3	4	0.1001	0.0679	− 0.2626	0.790	0.503
17	3	4	0.6800	0.3028	0.3235	1.943	1.500
18	3	4	0.9014	0.2755	− 0.1660	1.222	1.006

**Figure 11:**
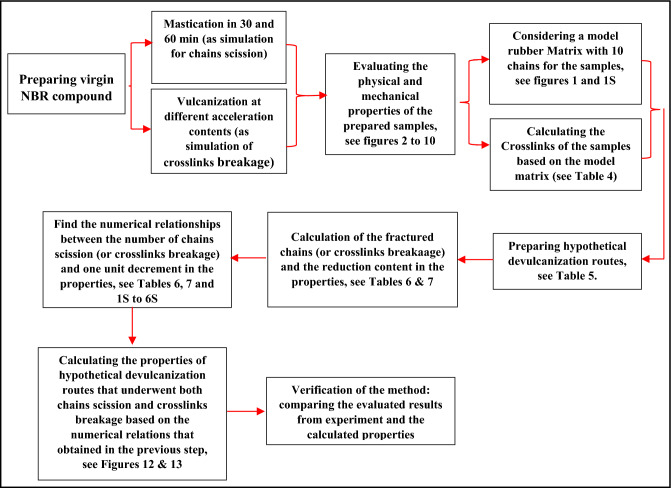
Flow chart of the prepared method for prediction of the decrement in the properties during devulcanization process.

Based on the results, it was found that there are direct numerical relationships between decrease in the hardness and modulus at 100% with the number of fractured chains. However, there are no clear relationships between derease of other properties and the number of chains scission. Figure 11 shows a flow chart for the prepared predicting method.

### Validation of the method

**Figure 12:**
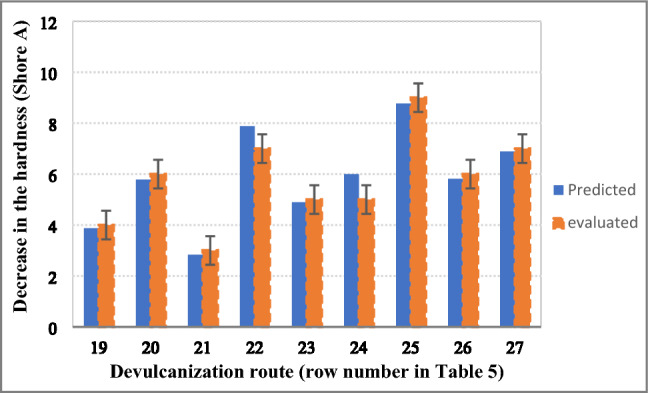
Relationship between the evaluated and predicted hardness for recycled samples at usual devulcanization condition.

**Figure 13:**
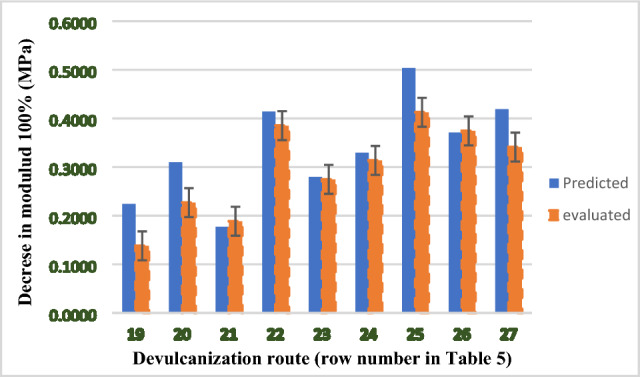
Relationship between evaluated and predicted modulus at 100% for recycled samples at usual devulcanization condition.

To validate the method (i.e. based on the relationships between the number of crosslinks breakage (CLB)/chains scission (CS) and decrement in the evaluated properties, the hardness and modulus at 100% of the usually devulcanized samples (i.e., rows 19–27 in Table 5) were calculated. It should be memorized that both crosslinks breakage and chains scission occurred in these hypothetical routes. Figures 12 and 13 show the evaluated contents of hardness and modulus at 100% against the predicted contents. It is clearly seen that the predicted contents are so close to the evaluated properties. Based on the findings, it was claimed that the method successfully predicted some properties that have direct correlations to the crosslinks breakage and/or chains scission.

## Conclusions

In this research, a facil method was prepared to predict the properties of devulcanized waste nitrile rubber, based on the relashionship between number of crosslinks breakage/chains scission and decline in the properties. For this purpose, nitrile rubber samples were prepared in three categories (i.e. different mastication times) and different crosslink densities (i.e. different CBS accelerator contents). The method was verified using comparing the calculated and evaluated properties for usually devulcanized samples. The following conclusions were obtained:

- It was found that curing properties (e.g. M_H_-M_L_ and CRI) have not direct correlation to chains scission/crosslinks breakage that happened during devulcanization process. on this basis, MDR results are not suitable for monitoring of the devulcanization performances.
- It was illustrated that evaluated crosslink density based on Flory-Rehner equation is a suitable criterion for evaluating performance of devulcanization process.
- Among the tensile properties, Young modulus and modulus at 100% showed direct relationship to crosslinks breakage and chain sscission (i.e. devulcanization content).
- A numerical correlation was obtained between decrease of the evaluated hardness and chains scission/crosslinks breakage that happened during devulcanization.
- In contrary to M_n_ and M_W_ values, M_Z_ and M_Z+1_ values showed a direct relationships to the number of crosslinks breakage and chains scission that occurred by devulcanization.
- The prepared mathematical method was used to predict the hardness and modulus at 100% of a series of devulcanized samples. The predicted (calculated ) data were very close to the evaluated hardness and modulus at 100% of the samples.

### Supplementary Information

Supplementary Information.

## Data Availability

All data generated or analysed during this study are included in this published article [and its supplementary information files].
