# Supplementary material for: Using a facile method to predict properties of recycled waste nitrile rubber (NBR) through devulcanization
Source: Sci Rep. 2023 Sep 16;13:15361. doi: 10.1038/s41598-023-42438-x (PMC10505169; doi:10.1038/s41598-023-42438-x)
Supplement: Supplementary file 1 — Supplementary Information. [file 41598_2023_42438_MOESM1_ESM.docx]

**Supporting Information**

**Using a Facile Method to Predict Properties of Recycled Waste Nitrile Rubber (NBR) Through Devulcanization**

**Mohammad Amin Ghowsi, Masoud Jamshidi^*^**

Constructional Polymers & Composites Research Lab., School of Chemical, Petroleum and Gas Engineering, Iran University of Science and Technology (IUST), Tehran, Iran.

* Corresponding author: Dr. Masoud Jamshidi, Associate professor, E-mail address: mjamshidi@iust.ac.ir, Tel. No.: ++98- 21-77240255, Fax No.: ++98-21-77240495.

**1S. Calculation of M_Z_ value**

According to the results of GPC analysis, the number of polymer molecules that could show the closest calculated average molecular weight to the evaluated ones were determined. Based on the try and error method, it was found that the calculated average molecular weight for 10 chains is the closest value to the evaluated one.

The Mz value was calculated as follows:

$M_{z}$=$\frac{\sum niMi^3}{\sum niMi^2}$ (1S)

Which n_i_ and M_i_ shows the number of chains and their molecular weight, respectively. Figure 1S shows the method used for calculation of M_Z_ value for the samples exposed to 1, 2 and 3 fractures in the middle of the chains.

**10 equal polymer chains with molecular weight of 1,006,537**

**11 polymer chains; molecular weight of 9 of them = 1,006,537 and molecular weight of two of them = 503,268.5**

**Calculated Mz= 1006537**

**Calculated Mz= 980049**

**12 polymer chains; molecular weight of 8 of them = 1,006,537 and molecular weight of four of them=503,268.5**

**13 polymer chains; molecular weight of 7 of them= 1,006,537 and molecular weight of 6 of them=503,268.5**

**Calculated Mz= 917725**

**Calculated Mz= 950618**

**Figure 1S.** Calculation of MZ value for a model sample contained 10 polymer molecules for virgin rubber compound and the compounds that had 1, 2 and 3 fractures in the middle.

**Table S1.** Changes in the properties in ideal devulcanization conditions.

| **Row no.** | **Number of CLB** | **Hardness** | | **Young’s Mod.** | | **100% Modulus** | | **300% Modulus** | | **Max Torque** | | **Min Torque** | |
| --- | --- | --- | --- | --- | --- | --- | --- | --- | --- | --- | --- | --- | --- |
|  |  | before | after | before | after | before | after | before | after | before | after | before | after |
|  |  | Shore A | | MPa | | MPa | | MPa | | dN.m | | dN.m | |
| 1 | 945 | 60 | 55 | 2.4444 | 1.4848 | 1.8411 | 1.4964 | 6.4195 | 5.6981 | 17.169 | 13.865 | 15.086 | 12.141 |
|  |  | Δ= 5 | | 0.9596 | | 0.3447 | | 0.7214 | | 3.3040 | | 2.9450 | |
| 2 | 593 | 58 | 55 | 2.1204 | 1.4848 | 1.7696 | 1.4964 | 6.7461 | 5.6981 | 14.856 | 13.865 | 13.347 | 12.141 |
|  |  | Δ= 3 | | 0.6356 | | 0.2732 | | 1.0480 | | 0.9910 | | 1.2060 | |
| 3 | 352 | 60 | 58 | 2.4444 | 2.1204 | 1.8411 | 1.7696 | 6.4195 | 6.7461 | 17.169 | 14.856 | 15.086 | 13.347 |
|  |  | Δ= 2 | | 0.3240 | | 0.0715 | | -0.3266 | | 2.3130 | | 1.7390 | |
| 4 | 916 | 57 | 53 | 2.3152 | 1.4694 | 1.6553 | 1.4560 | 6.6592 | 5.2667 | 17.457 | 14.009 | 15.374 | 12.213 |
|  |  | Δ= 4 | | 0.8458 | | 0.1993 | | 1.3925 | | 3.4480 | | 3.1610 | |
| 5 | 357 | 57 | 55 | 2.3152 | 2.0142 | 1.6553 | 1.5665 | 6.6592 | 6.2034 | 17.457 | 15.445 | 15.374 | 13.721 |
|  |  | Δ= 2 | | 0.3010 | | 0.0888 | | 0.4558 | | 2.0120 | | 1.6530 | |
| 6 | 881 | 56 | 51 | 1.5430 | 1.3847 | 1.5656 | 1.4285 | 6.5855 | 5.9607 | 18.391 | 14.655 | 16.092 | 12.644 |
|  |  | Δ= 5 | | 0.1583 | | 0.1371 | | 0.6248 | | 3.7360 | | 3.4480 | |
| 7 | 329 | 56 | 54 | 1.5430 | 1.4404 | 1.5656 | 1.4668 | 6.5855 | 6.4226 | 18.391 | 16.799 | 16.092 | 14.847 |
|  |  | Δ= 2 | | 0.1026 | | 0.0988 | | 0.1629 | | 1.5920 | | 1.2450 | |
| 8 | 559 | 55 | 53 | 2.0142 | 1.4694 | 1.5665 | 1.4560 | 6.2034 | 5.2667 | 15.445 | 14.009 | 13.721 | 12.213 |
|  |  | Δ= 2 | | 0.5448 | | 0.1105 | | 0.9367 | | 1.4360 | | 1.5080 | |
| 9 | 552 | 54 | 51 | 1.4404 | 1.3847 | 1.4668 | 1.4285 | 6.4226 | 5.9607 | 16.799 | 14.655 | 14.847 | 12.644 |
|  |  | Δ= 3 | | 0.0557 | | 0.0383 | | 0.4619 | | 2.1440 | | 2.2030 | |

| **Row no.** | **Number of chain scission (CS)** | **Number of CLB** | **Hardness** | | **Young’s Mod.** | | **100% Modulus** | | **300% Modulus** | | **Max Torque** | | **Min Torque** | |
| --- | --- | --- | --- | --- | --- | --- | --- | --- | --- | --- | --- | --- | --- | --- |
|  |  |  | before | after | before | after | before | after | before | after | before | after | before | after |
|  |  |  | Shore A | | MPa | | MPa | | MPa | | dN.m | | dN.m | |
| 10 | 1 | 0 | 53 | 51 | 1.4694 | 1.3847 | 1.4560 | 1.4285 | 5.2667 | 5.9607 | 14.009 | 14.655 | 12.213 | 12.644 |
|  |  |  | Δ= 2 | | 0.0847 | | 0.0275 | | -0.6940 | | -0.646 | | -0.431 | |
| 11 | 1 | 0 | 55 | 54 | 2.0142 | 1.4404 | 1.5665 | 1.4668 | 6.2034 | 6.4226 | 15.445 | 16.799 | 13.721 | 14.847 |
|  |  |  | Δ= 1 | | 0.5738 | | 0.0.0997 | | -0.2192 | | -1.354 | | -1.126 | |
| 12 | 1 | 0 | 57 | 56 | 2.3152 | 1.5430 | 1.6553 | 1.5656 | 6.6592 | 6.5855 | 17.457 | 18.391 | 15.374 | 16.092 |
|  |  |  | Δ= 1 | | 0.7722 | | 0.0897 | | 0.0737 | | -0.934 | | -0.718 | |
| 13 | 2 | 0 | 58 | 55 | 2.1204 | 2.0142 | 1.7696 | 1.5665 | 6.7461 | 6.2034 | 14.856 | 15.445 | 13.347 | 13.721 |
|  |  |  | Δ= 3 | | 0.1062 | | 0.2031 | | 0.5427 | | -0.589 | | -0.374 | |
| 14 | 2 | 0 | 55 | 53 | 1.4848 | 1.4694 | 1.4964 | 1.4560 | 5.6981 | 5.2667 | 13.865 | 14.009 | 12.141 | 12.213 |
|  |  |  | Δ= 2 | | 0.0154 | | 0.0404 | | 0.4314 | | -0.144 | | -0.072 | |
| 15 | 2 | 0 | 60 | 57 | 2.4444 | 2.3152 | 1.8411 | 1.6553 | 6.4195 | 6.6592 | 17.169 | 17.457 | 15.086 | 15.374 |
|  |  |  | Δ= 3 | | 0.1292 | | 0.1858 | | -0.2397 | | -0.288 | | -0.288 | |
| 16 | 3 | 0 | 55 | 51 | 1.4848 | 1.3847 | 1.4964 | 1.4285 | 5.6981 | 5.9607 | 13.865 | 14.655 | 12.141 | 12.644 |
|  |  |  | Δ= 4 | | 0.1001 | | 0.0679 | | -0.2626 | | -0.790 | | -0.503 | |
| 17 | 3 | 0 | 58 | 54 | 2.1204 | 1.4404 | 1.7696 | 1.4668 | 6.7461 | 6.4226 | 14.856 | 16.799 | 13.347 | 14.847 |
|  |  |  | Δ= 4 | | 0.6800 | | 0.3028 | | 0.3235 | | -1.943 | | -1.500 | |
| 18 | 3 | 0 | 60 | 56 | 2.4444 | 1.5430 | 1.8411 | 1.5656 | 6.4195 | 6.5855 | 17.169 | 18.391 | 15.086 | 16.092 |
|  |  |  | Δ= 4 | | 0.9014 | | 0.2755 | | -0.1660 | | -1.222 | | -1.006 | |

**Table S2.** Changes in the properties in worst devulcanization condition.

**Table 3S.** Correlation between hardness and chains scission for the worst devulcanization condition.

| **Row  no.** | **Number of CS** | **Decrease in**  **hardness**  (Shore A) | **Average decrease in**  **Hardness**  (Shore A) | **Average decrease in**  **hardness**  **per CS** (Shore A) |
| --- | --- | --- | --- | --- |
| 10 | 1 | 2 | 1.333 | 1.333 |
| 11 | 1 | 1 |  |  |
| 12 | 1 | 1 |  |  |
| 13 | 2 | 3 | 2.667 | 1.333 |
| 14 | 2 | 2 |  |  |
| 15 | 2 | 3 |  |  |
| 16 | 3 | 4 | 4 | 1.333 |
| 17 | 3 | 4 |  |  |
| 18 | 3 | 4 |  |  |

**Table 4S.** Correlation between modulus at 100% and chains scission for the worst devulcanization condition.

| **Row  no.** | **Number of CS** | **Decrease in modulus at 100%**  **(MPa)** | **Average decrease in modulus at 100% (MPa)** | **Average decrease in modulus at 100% per CS (MPa)** |
| --- | --- | --- | --- | --- |
| 10 | 1 | 0.0275 | 0.0723 | ≈ 0.072 |
| 11 | 1 | 0.0997 |  |  |
| 12 | 1 | 0.0897 |  |  |
| 13 | 2 | 0.2031 | 0.1431 | ≈ 0.072 |
| 14 | 2 | 0.0404 |  |  |
| 15 | 2 | 0.1858 |  |  |
| 16 | 3 | 0.0679 | 0.2154 | ≈ 0.072 |
| 17 | 3 | 0.3028 |  |  |
| 18 | 3 | 0.2755 |  |  |

**Table 5S.** Predicted hardness of the samples recycled through usual devulcanization condition (rows 19-27 in Table 5).

| **Row no.** | **Number of CS** | **Number of CLB** | **Decrease in hardness by  CS**  **(Shore A)** | **Decrease in hardness by CLB**  **(Shore A)** | **Predicted hardness ^a^**  **(Shore A)** | **Evaluated hardness**  **(Shore A)** | **Deviation**  **(Shore A)** |
| --- | --- | --- | --- | --- | --- | --- | --- |
| 19 | 1 | 642 | 1.333 | 3.433 ^b^ | 4.766 | 4 | +0.7 |
| 20 | 1 | 999 | 1.333 | 5.342 | 6.6 | 6 | +0.6 |
| 21 | 1 | 447 | 1.333 | 2.390 | 3.6 | 3 | +0.6 |
| 22 | 2 | 1095 | 2.667 | 5.856 | 8.5 | 7 | +1.5 |
| 23 | 2 | 536 | 2.667 | 2.866 | 5.5 | 5 | +0.5 |
| 24 | 2 | 743 | 2.667 | 3.973 | 6.6 | 5 | +1.6 |
| 25 | 3 | 1178 | 4 | 6.299 | 10.3 | 9 | +1.3 |
| 26 | 3 | 626 | 4 | 3.348 | 7.3 | 6 | +1.3 |
| 27 | 3 | 826 | 4 | 4.417 | 8.4 | 7 | +1.4 |

^a^ : Predicted hardness= Decrease in hardness by CS + Decrease in hardness by CLB

^b^ : Number of total CLB(642)/number of CLB needed for one unit reduction in hardness(187)=3.433

**Table 6S.** Predicted modulus at 100% of the samples recycled through usual devulcanization condition.

| **Row no.** | **Number of CS** | **Number of CLB** | **Decrease in modulus at 100% by CS**  **(MPa)** | **Decrease in modulus at 100% by CLB**  **(MPa)** | **Predicted Δ_modulus_ _at 100%_**  **(MPa)** | **Evaluated Δ_modulus_ _at 100%_**  **(MPa)** | **Deviation**  **(MPa)** |
| --- | --- | --- | --- | --- | --- | --- | --- |
| 19 | 1 | 642 | 0.0717 | 0.1544 | 0.2261 | 0.1380 | +0.0881 |
| 20 | 1 | 999 | 0.0717 | 0.2403 | 0.3120 | 0.2268 | +0.0852 |
| 21 | 1 | 447 | 0.0717 | 0.1075 | 0.1792 | 0.1885 | -0.0093 |
| 22 | 2 | 1095 | 0.1434 | 0.2633 | 0.4067 | 0.3851 | +0.0216 |
| 23 | 2 | 536 | 0.1434 | 0.1289 | 0.2723 | 0.2746 | -0.0023 |
| 24 | 2 | 743 | 0.1434 | 0.1787 | 0.3221 | 0.3136 | +0.0085 |
| 25 | 3 | 1178 | 0.2151 | 0.2833 | 0.4984 | 0.4126 | +0.0858 |
| 26 | 3 | 626 | 0.2151 | 0.1506 | 0.3657 | 0.3743 | -0.0086 |
| 27 | 3 | 826 | 0.2151 | 0.1987 | 0.4138 | 0.3411 | +0.0727 |

**2S. Calculation of devulcanization percent**

Figure 2S shows a hypothetical devulcanization for $M_{30}^{H}$ sample to $M_{60}^{L}$ sample.

**A hypothetical devulcanization**

$$\mathbf{M}_{\boldsymbol{30}}^{\mathbf{H}}$$

$$\mathbf{M}_{\mathbf{60}}^{\mathbf{L}}$$

**Figure 2S**. A hypothetical devulcanization process for $M_{30}^{H}$ sample.

The devulcanization percent for this route could be calculated as following:

Devulcanization percent (%)=$\frac{Number of crosslinks breakage}{Total crosslinks}\times100$ (1)

That based on the data of Table 5, devulcanization percent was calculated as follows:

$DP(\%$)$=\frac{Number of crosslinks(1)-Number of crosslinks(2)}{Number of crosslinks(1)}$ ×100 = $\frac{2929-1930}{2929} \times100=66.67$
